# Japanese gastric cancer treatment guidelines 2025 (7th edition)

**DOI:** 10.1007/s10120-025-01698-4

**Published:** 2026-01-22

**Authors:** Japanese Gastric Cancer Association

**Affiliations:** Japanese Gastric Cancer Association, 465 Kajii-cho, Kawaramachihirokoji, Kamigyo-ku, Kyoto, 602-0841 Japan

**Keywords:** Treatment guidelines, Surgery, Endoscopic resection, Chemotherapy, Evidence-based medicine

## Abstract

The 7th edition of the Gastric Cancer Treatment Guidelines was published in March 2025 covering new evidences. Following the format of the previous edition, this guideline consists of a text-based “Treatment” section and a “Clinical Questions” section that includes recommendations and explanations for clinical questions. The Treatment section describes the established standard treatments for gastric cancer, including surgery, endoscopic resection, and chemotherapy. The Clinical Questions section is based on a literature search and evaluation by the independent systematic review team. This allows for evidence-based responses to clinical issues and also presents future research topics in gastric cancer treatment.

## Preface to the English version

This English version is based on the Japanese version of the Japanese Gastric Cancer Treatment Guidelines, published as a book in 2025. However, this version reflects some of the new evidences that have emerged since the publication of the Japanese version. Some details of literature search and discussion that formed the basis of the recommendation for each clinical question has been given in text-book form in the original Japanese version of the guidelines, but has been omitted in the English version.

## Preface to the Japanese gastric cancer treatment guidelines 7th edition

The 7th edition of the Japanese Gastric Cancer Treatment Guidelines was completed in July 2025.

It presents appropriate treatment methods based on a detailed analysis of the vast amount of data on gastric cancer treatment accumulated in Japan and new evidence reported both domestically and internationally. Since the 5th edition, the guideline committee began to comply partially with the “Clinical Practice Guideline Development Manual” compiled by the Medical Information Network Distribution Service (Minds). Accordingly, in addition to the textbook-style description of standard treatments as seen in the previous editions, some clinical questions (CQs) were set and attempts were made to provide best possible answers though systematic review of literature.

In the current edition, while maintaining the portion written in the text-book style, the guideline committee referred more enthusiastically to the “Clinical Practice Guideline Development Manual 2020 ver. 3.0”. In brief, a greater number of CQs were set based on the latest advances in gastric cancer treatment, systematic reviews were conducted by the independent team, and the guideline committee decided the strengths of recommendation by consensus and interpretation of the systematic review. As a result, the basis for the recommended treatment was clearly presented, and areas where further research is needed to provide reliable recommended treatments were also clarified.

The main revisions to this edition are listed below.

1. The number of CQs on surgical treatment, endoscopic treatment, chemotherapy, and palliative care has been increased to 41 items.
2. Regarding perioperative chemotherapy, a CQ was prepared and recommendations were made jointly by the surgical and medical oncology committee members.
3. Three CQs were prepared regarding long-term impairment after gastrectomy, and recommendations were made.
4. CQs were prepared and recommendations were made regarding surgical treatment, endoscopic treatment, and chemotherapy in elderly patients.
5. Chemotherapy regimens for unresectable advanced/recurrent gastric cancer are listed in the algorithm in the “Treatment” section as “recommended regimens” and “conditionally recommended regimens.” The algorithm states that oxaliplatin combination regimens are preferable to cisplatin combination regimens.
6. Biomarkers (HER2, CLDN18, CPS, MSI/MMR) are explained in the “Treatment” section, and recommendations based on the latest research results are made in the CQs. These contents correspond to the “Guidelines for Biomarker Testing for Unresectable Advanced and Recurrent Gastric Cancer, version 1.1” formulated by the Japan Gastric Cancer Association.
7. CQs were created and recommendations were made regarding anamorelin in palliative treatment and palliative radiation therapy for hemorrhagic advanced gastric cancer.

## Treatments

### Treatment modalities and their indications

#### Algorithm of standard treatments to be recommended in clinical practice

**Fig. 1:**
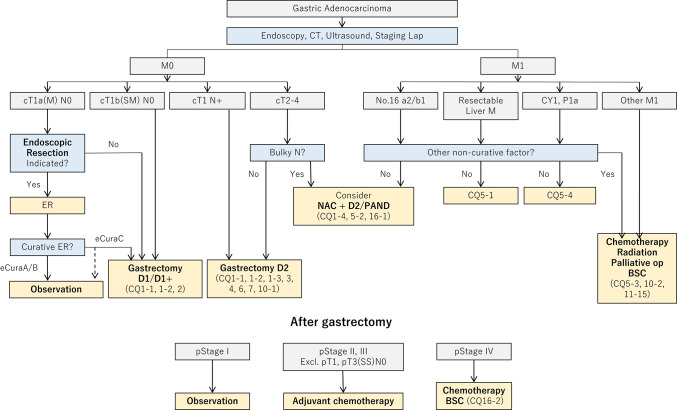
Algorithm of standard treatments. T/N/M and Stage are used in conjunction with the Japanese Classification of Gastric Carcinoma 15th edition [1] and TNM classification 8th edition [2]

The algorithm is shown in Fig. 1. Description of the tumor status (T/N/M and stage) in this edition is based on the 15th edition of the Japanese Classification of Gastric Carcinoma [1], which is identical to the 8th edition of the International Union Against Cancer (UICC)/TNM Classification [2].

#### Summary of T, N, and M categories and stage grouping based on the 15th edition of the Japanese classification of gastric carcinoma [1]

N grade is categorized according to the number of metastatic lymph nodes among the regional lymph nodes (No. 1–12. 14v); N1: 1–2, N2: 3–6, N3a: 7–15, N3b: ≥ 16.

M1: metastasis to the distant organs, peritoneal surface (including CY1), and lymph　nodes outside the regional lymph nodes.

Stage grouping: See Table 1.

**Table 1:**
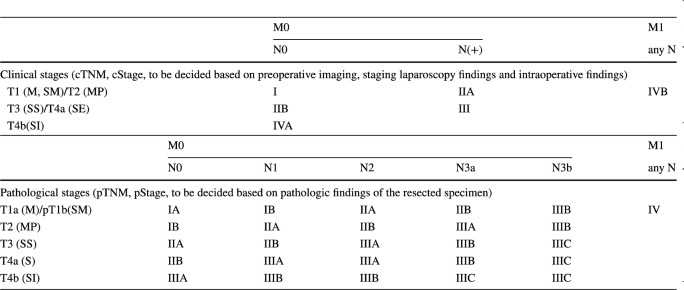
Stage grouping

## Surgery

### Types and definitions of gastric surgery

#### Standard gastrectomy and non-standard gastrectomy in surgery with curative intent

##### Standard gastrectomy

**Fig. 2:**
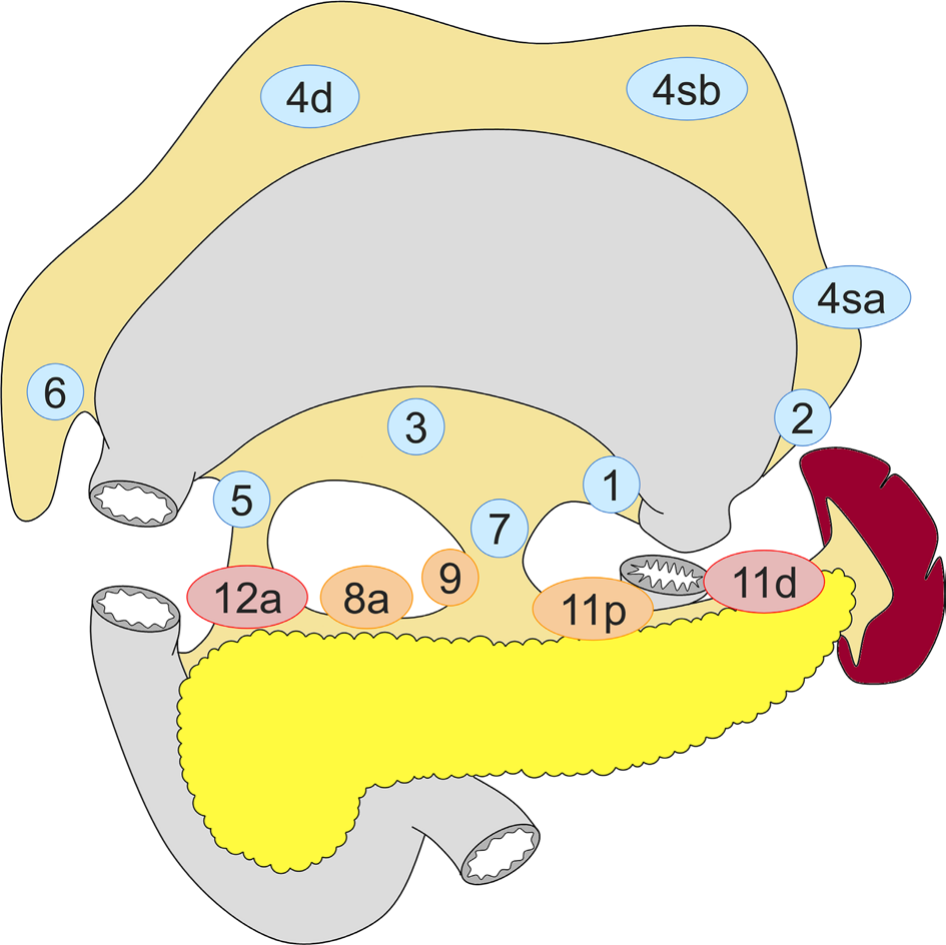
Lymph node dissection in total gastrectomy. Lymph node stations in blue need to be dissected in D1 dissection. In addition, lymph node stations in orange need to be dissected in D1 + dissection, and lymph node stations in red need to be dissected as well in D2 dissection

**Fig. 3:**
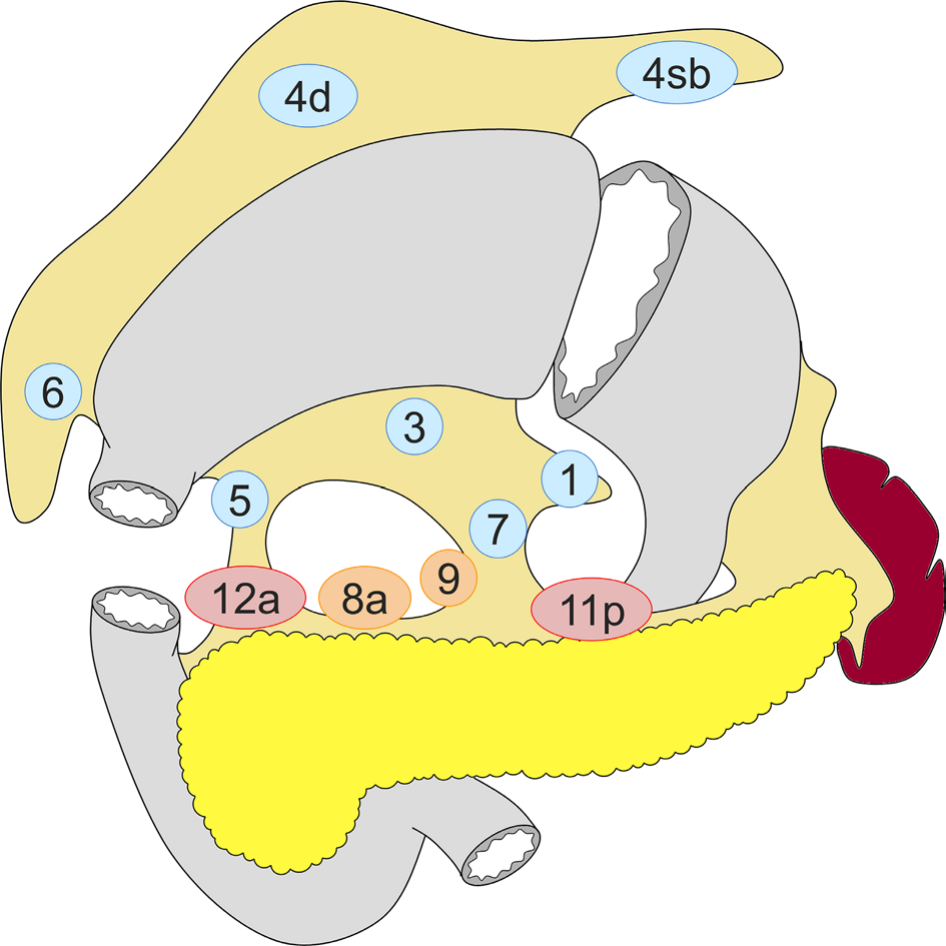
Lymph node dissection in distal gastrectomy. Lymph node stations in blue need to be dissected in D1 dissection. In addition, lymph node stations in orange need to be dissected in D1 + dissection, and lymph node stations in red need to be dissected as well in D2 dissection

**Fig. 4:**
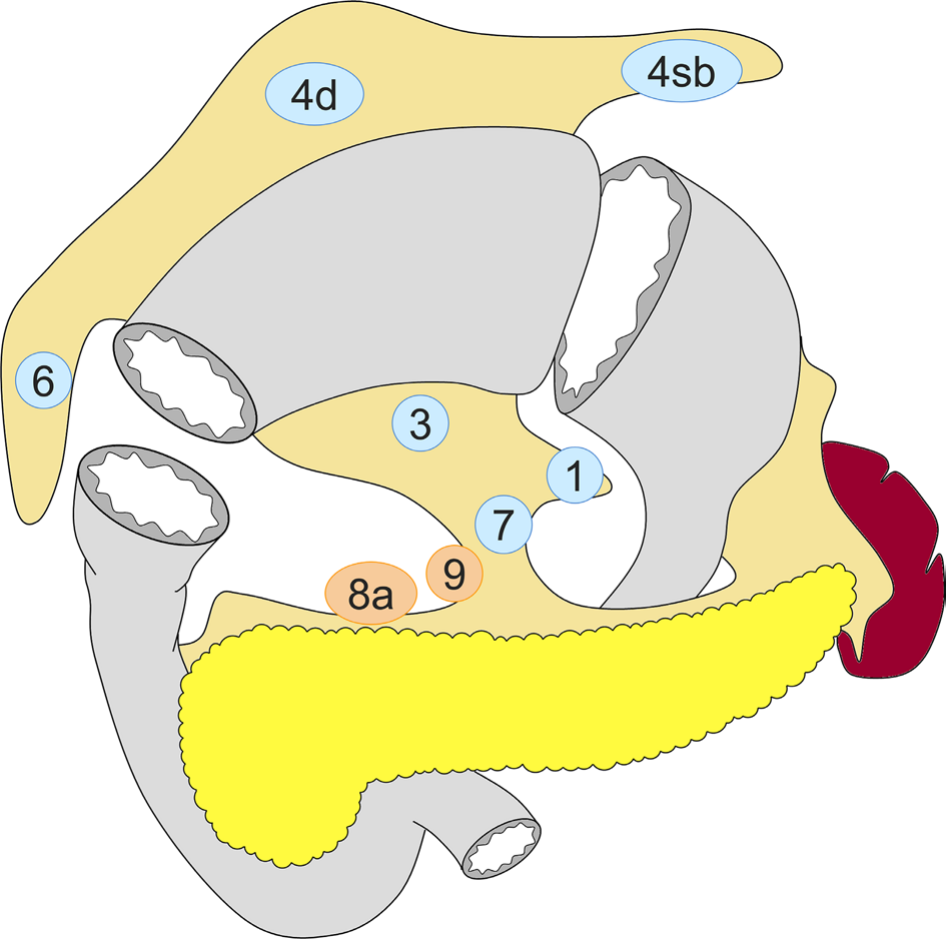
Lymph node dissection in pylorus-preserving gastrectomy. Lymph node stations in blue need to be dissected in D1 dissection. In addition, lymph node stations in orange need to be dissected in D1 + dissection

**Fig. 5:**
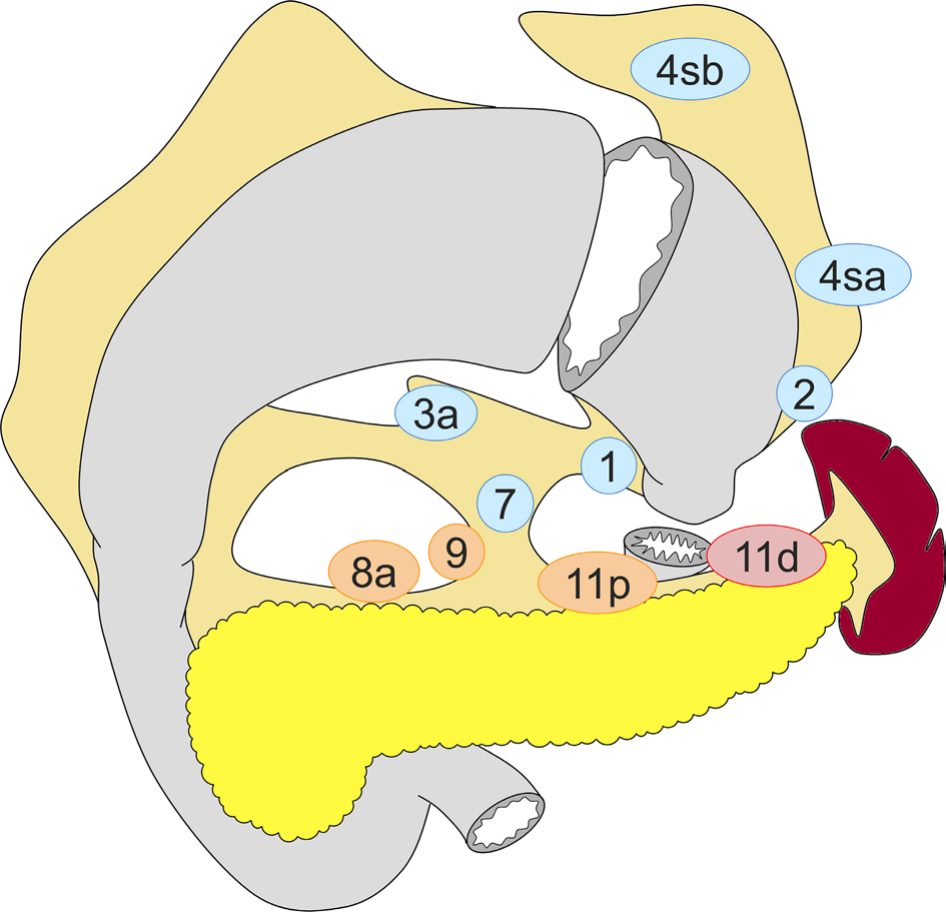
Lymph node dissection in proximal gastrectomy. Lymph node stations in blue need to be dissected in D1 dissection. In addition, lymph node stations in orange need to be dissected in D1 + dissection, and lymph node stations in red need to be dissected as well in D2 dissection

Standard gastrectomy is the principal surgical procedure performed with curative intent. It involves resection of at least two-thirds of the stomach with D2 lymph node dissection (refer to the section on “lymph node dissection” and Figs. 2, 3, 4 and 5 for the definition of D-categories).

##### Non-standard gastrectomy

In non-standard gastrectomy, the extent of gastric resection and/or lymphadenectomy is determined depending on tumor stage. It includes modified surgery and extended surgery.

- Modified surgery

The extent of gastric resection and/or lymphadenectomy is decreased (D1, D1 + , etc.) compared with standard surgery.

- Extended surgery
  1. Gastrectomy with combined resection of adjacent involved organs.
  2. Gastrectomy with extended lymphadenectomy exceeding D2.

#### Non-curative surgery

Non-curative surgery is offered to patients who are considered incurable. It can be semi-classified into either palliative surgery or reduction surgery depending on the aim of surgery.

##### Palliative surgery

Serious symptoms such as bleeding or obstruction may develop in a patient with advanced/metastatic gastric cancer. Surgery to relieve imminent symptoms may then be considered an option, and palliative gastrectomy or gastrojejunostomy is selected depending on the resectability of the primary tumor and/or surgical risks. Surgical intervention for patients with gastric outlet obstruction has been reported to result in improvement of oral intake [3] and maintenance of quality of life (QOL) which reportedly leads to improvements in prognosis [4].

##### Reduction surgery

Reduction surgery is defined as gastrectomy performed for patients with incurable factors such as unresectable liver metastasis and peritoneal metastasis, with no tumor-associated symptoms such as bleeding, obstruction, and pain. It aims to prolong survival or to delay the onset of symptoms by reducing tumor volume. However, an international, cooperative, randomized, controlled trial (REGATTA, JCOG0705/KGCA01) failed to prove a survival benefit of reduction surgery [5]. Therefore, surgeons are strongly advised not to perform this type of surgery for a patient for whom systemic chemotherapy is tolerable.

##### Conversion surgery

Conversion surgery is defined as surgery performed with curative intention for initially unresectable cases who are converted to be resectable by the efficacy of systemic therapy. An international, multicenter, retrospective, observational study (CONVO-GC-1) conducted jointly by Japan, Korea, and China demonstrated that favorable long-term survival was achieved when R0 surgery was performed, suggesting the potential efficacy of conversion surgery. However, there have been no randomized, controlled trials comparing the continuation of chemotherapy versus gastrectomy after chemotherapy response, and the efficacy of conversion surgery remains unclear. Results from the ongoing clinical trial (JCOG2301) conducted by the Japanese Clinical Oncology Group are awaited.

### Extent of gastric resection

#### Surgery for gastric cancer

Surgery for gastric cancer is defined as follows in order of the stomach volume to be resected.

- *Total gastrectomy* Total resection of the stomach including the cardia and pylorus.
- *Subtotal gastrectomy* Stomach resection on the pyloric side by cutting off the lesser curvature along almost its entire length and partially cutting off the short gastric artery.
- *Distal gastrectomy* Stomach resection including the pylorus. The cardia is preserved. In the standard gastrectomy, two-thirds of the stomach is resected.
- *Pylorus*-*preserving gastrectomy* (*PPG*) Stomach resection preserving the upper third of the stomach and the pylorus along with a portion of the antrum.
- *Proximal gastrectomy* (*PG*) Stomach resection including the cardia (esophagogastric junction). The pylorus is preserved.
- *Segmental gastrectomy* Circumferential resection of the stomach preserving the cardia and pylorus.
- *Local resection* Non-circumferential resection of the stomach.
- *Non*-*resectional surgery* Bypass surgery, gastrostomy, jejunostomy.

In addition, surgery for cancer of the gastric remnant is defined as follows.

- *Completion gastrectomy* Total resection of the remnant stomach including the cardia or pylorus depending on the type of previous gastrectomy.
- *Subtotal resection of remnant stomach* Distal resection of the remnant stomach preserving the cardia.

#### Determination of the extent of gastric resection

##### Resection margin

A sufficient resection margin should be ensured when determining the resection line in gastrectomy with curative intent. A proximal margin of at least 3 cm is recommended for T2 or deeper tumors with an expansive growth pattern (types 1 and 2) and 5 cm for those with an infiltrative growth pattern (types 3 and 4). When these rules cannot be satisfied, it is advisable to examine the whole thickness of the proximal resection margin by frozen section. For tumors invading the esophagus, a resection margin > 5 cm is not necessarily required, but frozen section examination of the resection line is preferable to ensure an R0 resection.

For T1 tumors, a gross resection margin of 2 cm should be obtained. When the tumor border is unclear, and difficulties in determining the resection line are expected, preoperative endoscopic marking of the tumor border by clips based on the biopsy results would be helpful.

##### Selection of gastrectomy

The standard surgical procedure for clinically node-positive (cN +) or T2–T4a tumors is either total or distal gastrectomy. Distal gastrectomy is selected when a satisfactory proximal resection margin (see above) can be obtained. When obtaining a clean proximal resection margin is not possible, total gastrectomy is selected. Even in a case in which a satisfactory proximal resection margin can be obtained, pancreatic invasion by tumor requiring pancreaticosplenectomy necessitates total gastrectomy regardless of tumor location. Total gastrectomy with splenectomy should be considered for tumors that are located along the greater curvature. For adenocarcinoma of the esophagogastric junction, proximal gastrectomy is also considered (CQ6-2).

For cT1N0 tumors, the following types of gastric resection can be considered according to tumor location.

- *Pylorus-preserving gastrectomy (PPG)* for tumors in the middle portion of the stomach with the distal tumor border at least 4 cm proximal from the pylorus (CQ2-1).
- *Proximal gastrectomy* for proximal tumors in which more than half of the distal stomach can be preserved (CQ2-2).
- *Subtotal gastrectomy (sTG)* for tumors in the upper third of the stomach in which proximal stomach can be preserved even if it is extremely small (CQ2-3).
- Local resection of the stomach and segmental gastrectomy should still be regarded as investigational treatments.

### Lymph node dissection

#### Extent of lymph node dissection

The extent of lymphadenectomy is classified by the D-level criteria into D1, D1 + , or D2, and is defined as follows according to the type of gastrectomy conducted. The indications for each of the D levels are described in the subsequent section. See the descriptions under the title “Esophagogastric junctional cancer” for the current recommendations on the extent of lymph node dissection for esophagogastric junctional carcinoma.

#### Definition of the D levels

The extent of systematic lymphadenectomy is defined as follows, according to the type of gastrectomy conducted. When the extent of lymphadenectomy performed does not fully comply with the D-level criteria, the lymph node station that has been additionally resected or left in situ could be recorded as in the following examples: D1 (+ No. 8a), D2 (− No. 12a). However, when entering data in the nationwide database, the D levels need to be strictly determined and should be downgraded when omitting resection of any of the lymph node stations that should have been resected to fulfill the criteria of a certain D level. (e.g., D2 (− No. 12a) should be entered as D1 +).

#### Total gastrectomy (Fig. 2)

D0: Lymphadenectomy less than D1.

D1: Nos. 1–7.

D1+: D1 + Nos. 8a, 9, 11p.

D2: D1 + Nos. 8a, 9, 11p, 11d, 12a.

For tumors invading the esophagus, resection of Nos. 19, 20, and 110* should be added to D2.

#### Distal gastrectomy (Fig. 3)

D0: Lymphadenectomy less than D1.

D1: Nos. 1, 3, 4sb, 4d, 5, 6, 7.

D1+: D1 + Nos. 8a, 9.

D2: D1 + Nos. 8a, 9, 11p, 12a.

#### Pylorus-preserving gastrectomy (Fig. 4)

D0: Lymphadenectomy less than D1.

D1: Nos. 1, 3, 4sb, 4d, 6**, 7.

D1+: D1 + Nos. 8a, 9.

#### Proximal gastrectomy (Fig. 5)

D0: Lymphadenectomy less than D1.

D1: Nos. 1, 2, 3a, 4sa, 4sb, 7.

D1+: D1 + Nos. 8a, 9, 11p.

D2: D1 + Nos. 8a, 9, 11p, 11d.

For tumors invading the esophagus, Nos. 19, 20, and 110* should additionally be dissected in D2.

*No. 110 lymph nodes (lower thoracic para-esophageal nodes) in gastric cancer invading the esophagus are those attached to the lower part of the esophagus that is removed to obtain a sufficient resection margin.

**D level should not be changed in cases in which No. 6i was incompletely dissected in pylorus-preserving gastrectomy.

#### Indications for lymph node dissection

In principle, D2 lymphadenectomy is indicated for cN + or ≥ cT2 tumors, and D1 or D1 + lymphadenectomy is indicated for cT1N0 tumors. Since pre- and intraoperative diagnoses regarding the depth of tumor invasion and nodal involvement remain unreliable, D2 lymphadenectomy should be performed whenever the possibility of nodal involvement cannot be dismissed.

##### D1 lymphadenectomy

D1 lymphadenectomy is indicated for cT1a tumors that do not meet the criteria for endoscopic mucosal resection (EMR)/endoscopic submucosal dissection (ESD), and for cT1bN0 tumors that are histologically of differentiated type and 1.5 cm or smaller in diameter.

##### D1 + lymphadenectomy

D1 + lymphadenectomy is indicated for cT1N0 tumors other than the above.

##### D2 lymphadenectomy

D2 lymphadenectomy is indicated for potentially curable cT2–T4 tumors, as well as cT1N + tumors. The spleen should be preserved in total gastrectomy for advanced cancer of the proximal stomach provided the tumor does not involve the greater curvature [6] (CQ3-2).

##### D2 + lymphadenectomy

Gastrectomy with extended lymphadenectomy beyond D2 is classified as a non-standard gastrectomy, and could be considered for the following cases, although hard evidence is lacking, on the condition that it can be conducted safely.

- Dissection of No. 10 (splenic hilar lymph nodes) with or without splenectomy for cancer of the proximal stomach invading the greater curvature (D2 + No. 10) (CQ3-2).
- Dissection of No. 14v (superior mesenteric venous lymph node) for cancer of the distal stomach with metastasis to the No. 6 lymph nodes (D2 + No. 14v).
- Dissection of No. 12b (in the hepatoduodenal ligament along the bile duct), No. 12p (in the hepatoduodenal ligament along the portal vein), and No. 13 (posterior head of the pancreas lymph node) for cancer invading the duodenum (D2 + No. 13) [7, 8]. Metastases to the Nos. 12b, 12p, and 13 nodes, which are not included in the regional lymph nodes for gastric cancer, should usually be classified as M1. However, since the Nos. 12b, 12p, and 13 nodes are among the regional lymph nodes for cancer of the duodenum according to the TNM classification and the Japanese Classification of Gastric Carcinoma 15th edition, these should be regarded as regional lymph nodes once gastric cancer invades the duodenum.
- Dissection of No. 16 (abdominal aortic lymph node) after neoadjuvant chemotherapy for cancer with extensive lymph node involvement (D2 + No. 16) (CQ5-1).

### Esophagogastric junctional cancer

**Fig. 6:**
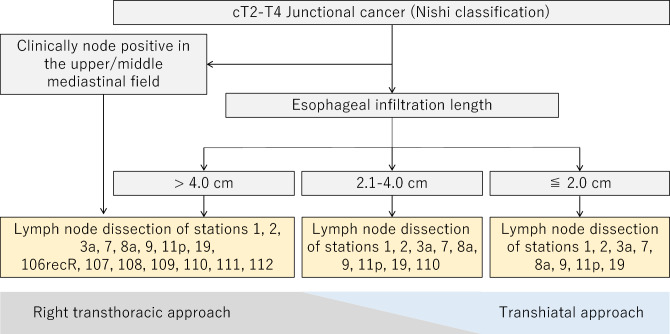
Algorithm of the surgical approach and lymph node dissection for esophagogastric junctional carcinoma

The current edition of the Japanese Gastric Cancer Treatment Guidelines defines the extent of lymphadenectomy according to the type of gastrectomy, regardless of tumor location. However, only for esophagogastric junctional cancer, either adenocarcinoma or squamous cell carcinoma, of which the center is located within 2 cm of the esophagogastric junction, there is no consensus on the type of resection and the extent of lymphadenectomy as a standard of care for this category. The Japanese Gastric Cancer Association and Japan Esophageal Society joined forces to conduct a prospective study of esophagogastric junctional cancer of cT2-T4, and the incidences of lymph node metastasis were examined [9]. It was found that the incidence of lymph node metastasis differed according to the length of esophageal invasion; a low incidence of mediastinal lymph node metastasis in tumors with esophageal invasion shorter than 2 cm, especially less than 1 cm; a high incidence of lower mediastinum lymph nodes (No. 110), but a low incidence of upper and middle mediastinum lymph nodes in tumors with esophageal invasion of 2.1–4.0 cm; and a high incidence of both upper and middle mediastinum lymph nodes in tumors with esophageal invasion greater than 4 cm. Based on these results, Fig. 6 shows the approach and algorithm for dissecting regional lymph nodes with a probability of metastasis of 10% or more (Fig. 6). Although it has not been confirmed, because no survival results have been obtained, it seems reasonable to follow this algorithm for the treatment of esophageal junction carcinoma of cT2 or deeper at present.

#### Extent of the resection of the esophagus and stomach

One of the following procedures is selected for esophagogastric junctional cancer: proximal gastrectomy with or without lower esophageal resection; total gastrectomy with or without lower esophageal resection; or esophageal resection and proximal gastrectomy (CQ6-2).

#### Extent of lymphadenectomy (CQ6-1)

Although long-term results for the survival benefit of lymphadenectomy following the algorithm have not yet been obtained, it seems reasonable to follow this algorithm for the treatment of esophagogastric junctional cancer of cT2 or deeper. Nevertheless, the guidelines currently do not make either more extensive or limited lymphadenectomies inappropriate. However, even in tumors with esophageal invasion of 2 cm or less, surgeons are expected to dissect part of No. 110 lymph nodes that are attached to the portion of esophagus resected to obtain a sufficient resection margin. The definition of D level follows surgery for gastric carcinoma with esophageal invasion, but if the length of esophageal invasion exceeds 4 cm, D level should be classified according to the esophagogastric junction cancer in the Japanese Classification of Esophageal Cancer.

### Miscellaneous

#### Vagal nerve preservation

It has been reported that preservation of the hepatic branch of the anterior vagus and/or the celiac branch of the posterior vagus contributes to improving postoperative QOL through reducing post-gastrectomy gallstone formation, diarrhea, and/or weight loss.

#### Omentectomy

Removal of the greater omentum is usually integrated in the standard gastrectomy for T3 or deeper tumors. For T1/T2 tumors, the omentum more than 3 cm away from the gastroepiploic artery may be preserved (CQ3-1). A clinical trial confirming the non-inferiority of omentum preservation to omentectomy for T3 or deeper tumors (JCOG1711) is now underway.

#### Bursectomy

Bursectomy for tumors penetrating the serosa of the posterior gastric wall had been performed with the aim of removing microscopic tumor deposits within the omental cavity. However, the survival benefit of this procedure has been refuted by a large-scale, randomized trial (JCOG1001) [10].

#### Combined resection of adjacent organ(s)

For tumors in which the primary or metastatic lesion directly invades adjacent organs, combined resection of the involved organ may be performed to obtain an R0 resection. Even in cases of invasion of the duodenum and head of the pancreas, pancreaticoduodenectomy is weakly recommended when the patient is in good general condition, lymph node metastasis is relatively mild, and R0 resection is possible (CQ3-3).

#### Approaches to the lower esophagus

A transhiatal abdominal approach has been recommended for gastric cancers invading less than 3 cm of the distal esophagus based on the results of the JCOG9502 trial, and a transthoracic approach has been considered when a greater length of esophagus is involved [11]. The results of a recently reported cooperative study by the Japan Gastric Cancer Association and the Japanese Esophageal Society suggest that dissection of the upper and middle mediastinum might be omitted when the length of esophageal invasion is 4 cm or less. Therefore, a transhiatal abdominal approach can be recommended for cases in which the length of esophageal invasion is 4 cm or less, if safe excision and reconstruction are technically possible.

#### Laparoscopic surgery

The non-inferiority of laparoscopic distal gastrectomy for clinical stage I gastric cancer to open distal gastrectomy was confirmed in phase 3, randomized, controlled, clinical trials conducted in Japan and Korea (JCOG0912, KLASS-01) [12, 13]. Therefore, laparoscopic distal gastrectomy for clinical stage I gastric cancer is strongly recommended as one of the standard treatment options. The feasibility of laparoscopy-assisted total or proximal gastrectomy has also been confirmed in a single-arm, confirmatory, clinical trial (JCOG1401) [14]. Since survival outcomes have also been reported [15], this guideline strongly recommends performing this procedure (CQ1-1).

For advanced gastric cancer, large-scale, randomized, clinical trials confirming safety and long-term survival of laparoscopic distal gastrectomy have been conducted in Japan, Korea, and China (JLSSG0901, KLASS-02, CLASS-01) [16–18]. Safety analyses showed that no increase in complications after surgery was observed with the laparoscopic approaches. The non-inferiority of overall survival of laparoscopic distal gastrectomy to open distal gastrectomy has also been confirmed [19–21]. This guideline strongly recommends performing laparoscopic distal gastrectomy for advanced gastric cancer of Stage II or more (CQ1-1). Regarding total gastrectomy, several retrospective studies have shown no significant differences in survival or postoperative complications compared with open surgery; therefore, this guideline weakly recommends laparoscopic total gastrectomy (CQ1-3). The results of an ongoing randomized, clinical trial (KLASS-06) are awaited.

All laparoscopic procedures must be performed by or under the guidance of a surgeon who is either qualified by the endoscopic surgical skill system of the Japanese Society of Endoscopic Surgery or acknowledged as having acquired equivalent skills.

#### Robotic surgery

Robotic surgery has been incorporated by numerous surgical teams and is reputed for its advantage particularly when attempting technically complex surgery. In Japan, it has been shown to decrease the incidence of surgical complications when compared with a similar group of patients who were treated by the laparoscopic approach. This observation led to the approval in 2018 of health insurance coverage for robot-assisted gastrectomy. However, since those studies were based on a single-arm trial or retrospective comparison, a clear benefit of robotic gastrectomy has not been demonstrated [19, 20, 22, 23]. A randomized, controlled study (JCOG1907) to confirm the superiority of robotic gastrectomy to laparoscopic gastrectomy in terms of reducing morbidity for clinical T1-4a N0-2 gastric cancer is ongoing [24]. At present, robotic surgery for resectable gastric cancer is weakly recommended. For robotic gastrectomy, it is necessary to fulfil the standard quality criteria for the surgeon and the facility (CQ1-2).

### Reconstruction after gastrectomy

The following reconstruction methods are usually used. Each has advantages and disadvantages. The functional benefits of pouch reconstruction are yet to be established.

#### Total gastrectomy

- Roux-en-Y esophagojejunostomy.
- Jejunal interposition.
- Double-tract method.

#### Distal gastrectomy

- Billroth I gastroduodenostomy.
- Billroth II gastrojejunostomy.
- Roux-en-Y gastrojejunostomy.
- Jejunal interposition.

#### Pylorus-preserving gastrectomy

- Gastro-gastrostomy.

#### Proximal gastrectomy

- Esophagogastrostomy.
- Jejunal interposition.
- Double-tract method.

## Endoscopic resection

### Methods of endoscopic resection

#### Endoscopic mucosal resection (EMR)

The lesion, together with the surrounding mucosa, is lifted by submucosal injection of fluid cushions such as normal saline and removed using a high-frequency steel snare [25, 26].

#### Endoscopic submucosal dissection (ESD)

The mucosa surrounding the lesion is circumferentially incised using a high-frequency electric device such as an insulation-tipped knife, and the submucosal layer is dissected from the muscularis propria layer [27–29].

### Handling of endoscopically resected specimens

#### Handling of resected specimens

The resected specimens should be handled according to the rules described in the Japanese Classification of Gastric Carcinoma 15th edition [1].

#### Definitions of differentiated-type and undifferentiated-type carcinomas

The tumor biopsy specimens and endoscopically resected tumors are classified histologically into either the differentiated type or the undifferentiated type. The former includes malignant epithelial tumor, general type, of papillary adenocarcinoma (pap) and tubular adenocarcinoma (tub1, tub2), and the latter includes that of poorly differentiated adenocarcinoma (por1, por2) and signet ring cell carcinoma (sig) according to the Japanese Classification of Gastric Carcinoma 15th edition.

When mucinous adenocarcinoma (muc) is found at the submucosal layer, the resected specimen is handled as undifferentiated type, regardless of whether it is considered to derive from the differentiated or undifferentiated type.

#### Histological predominance and intratumoral ulcerative findings (UL)

A tumor consisting of components of both differentiated-type and undifferentiated-type carcinoma is, nevertheless, classified into one of the two types according to quantitative predominance. In addition, when more than one histological type is found in a tumor, all histological types are to be recorded in the order of quantitative predominance, e.g., tub2 > tub1. The diagnosis of UL1 is principally made based on the histological evidence of ulcerative findings. However, the histological diagnosis of UL is sometimes difficult because of a biopsy-derived scar. Thus, endoscopic and/or radiological evidence should also be taken into account when making a conclusive diagnosis. A biopsy-derived scar is usually observed histologically as fibrosis restricted to small areas just beneath the muscularis mucosae [30]. If it cannot be discriminated from the ulcer scar, it should be classified as UL1.

### Indications for endoscopic resection (CQ17)

Lesions that could technically be resected by endoscopy are classified into the following three categories depending on the strength of evidence. “A tumor indicated for endoscopic resection as a standard treatment (absolute indication)” is defined as a tumor in which the possibility of harboring lymph node metastasis is less than 1%. For this population, endoscopic resection is expected to have a therapeutic effect equivalent to surgical resection. “A tumor indicated for endoscopic resection as an investigational treatment (expanded indication)” is defined as a tumor for which sufficient evidence for the long-term outcome after endoscopic resection is lacking, although the possibility of harboring lymph node metastasis is less than 1%. “A tumor indicated for endoscopic resection as clinical practice under some circumstances (relative indication)” is defined as a tumor that would usually be treated by surgical resection, but for which endoscopic resection may still lead to cure and could, therefore, be an option when surgery cannot be recommended due to various clinical circumstances.

#### Principles for indications

Endoscopic resection is considered for tumors that have a very low possibility of lymph node metastasis and are suitable for en bloc resection [31, 32].

#### Indications

#### Absolute indication

*Absolute indication for EMR or ESD* [33–35]

A differentiated-type adenocarcinoma without ulcerative findings (UL0), in which the depth of invasion is clinically diagnosed as T1a, and the diameter is ≤ 2 cm.

*Absolute indication for ESD*

- A differentiated-type adenocarcinoma without ulcerative findings (UL0), in which the depth of invasion is clinically diagnosed as T1a, and the diameter is > 2 cm.
- A differentiated-type adenocarcinoma with ulcerative findings (UL1), in which the depth of invasion is clinically diagnosed as T1a, and the diameter is ≤ 3 cm.
- An undifferentiated-type adenocarcinoma without ulcerative findings (UL0), in which the depth of invasion is clinically diagnosed as T1a, and the diameter is ≤ 2 cm.

##### Expanded indication [36]

- A locally recurrent lesion, in which the depth of invasion is clinically diagnosed as T1a, after initial endoscopic resection of endoscopic curability (eCura) C-1 described below for a lesion with an absolute indication and differentiated-type adenocarcinoma

#### Relative indication

A standard therapy is surgical resection for tumors that do not fulfill the absolute or expanded indications. However, endoscopic resection could be an option for elderly and high-operative-risk patients with severe comorbidities. Such cases are considered relative indications, and endoscopic resection could be performed, provided that consent is obtained from the patient after explaining the risk of residual disease in the form of lymph node metastasis.

### Curability of endoscopic resection

#### Evaluation of curability

Two factors should be considered for curability assessment: completeness of the primary tumor removal and possibility of lymph node metastasis.

#### Endoscopic curability A (eCuraA)

The resection is classified as endoscopic curability A (eCuraA) when all of the following conditions are fulfilled, provided the cancer has no ulcerative findings (UL0) and is en bloc resected: i) any tumor size in case of histologically differentiated type-dominant, or ii) tumor size ≤ 2 cm in case of histologically undifferentiated type-dominant, pT1a, negative horizontal margin (HM0), negative vertical margin (VM0), and no lymphovascular infiltration (Ly0, V0).

When the cancer has ulcerative findings (UL1), the resection is still classified as eCuraA when all of the following conditions are fulfilled: iii) en bloc resection, tumor size ≤ 3 cm, histologically differentiated type-dominant, pT1a, HM0, VM0, Ly0, V0.

Based on the recent report of long-term results of a prospective cohort study [37], resections fulfilling the following conditions are also classified as eCuraA: iv) tumor size ≤ 3 cm in case of histologically differentiated type-dominant, pT1b (SM1; submucosal invasion < 500 μm from the muscularis mucosae), HM0, VM0, Ly0, V0. However, if the undifferentiated component exceeds 2 cm in length in i), or if undifferentiated components are present in the invasion to SM in iv), the endoscopic curability is classified as C-2 (eCuraC-2) [38].

#### Endoscopic curability B (eCuraB)

The dissection of the lesion as an “expanded indication” is classified as endoscopic curability B (eCuraB) when the criteria for eCuraA iii) and iv) are fulfilled: sum of the size of locally recurrent lesion and initial ESD specimen ≤ 30 mm [39].

#### Endoscopic curability C (eCuraC)

The resection is classified as endoscopic curability C (eCuraC) when it does not fulfill the conditions described above to be classified as either eCuraA or eCuraB.

The resection is classified as endoscopic curability C-1 (eCuraC-1) when it is histologically differentiated type either not resected en bloc or had a positive horizontal margin even though fulfilling other criteria to be classified as either eCuraA or eCuraB. All other eCuraC resections are subclassified as endoscopic curability C-2 (eCuraC-2).

### Management after endoscopic resection (Fig. 7)

Treatments should be planned as follows after evaluation of curability based on the histological examination of the resected specimens.

**Fig. 7:**
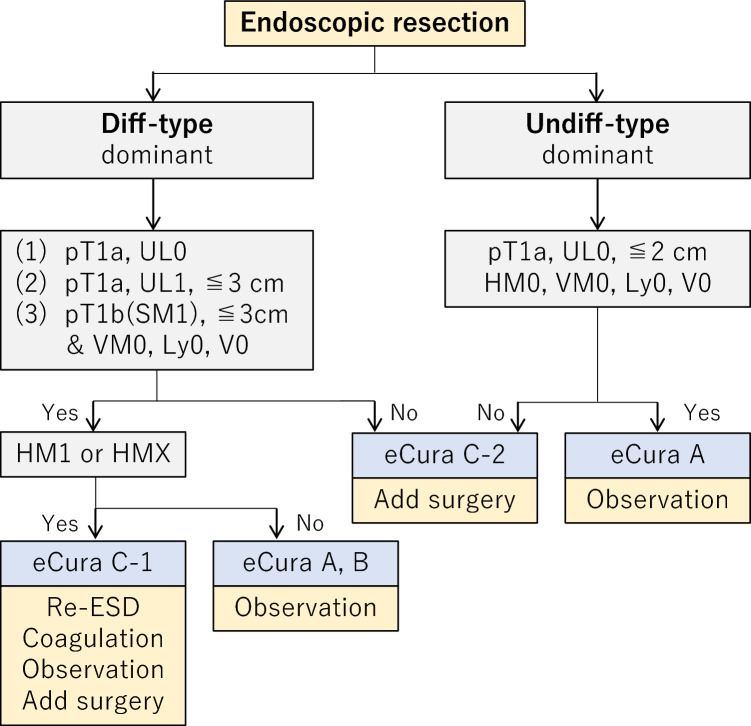
Algorithm showing the curability decision and additional treatments for patients who have undergone endoscopic resection

#### Treatments after eCuraA or eCuraB

Follow-up with annual endoscopy is recommended after eCuraA resection [40]. Follow-up with annual or biannual endoscopy and abdominal ultrasonography or computed tomography (CT) for surveillance of metastases is recommended after eCuraB resection [41]. For both eCuraA and eCuraB resections, it has been recommended that examinations for *Helicobacter pylori* be performed, and that if positive, it should be eradicated [42–44]. Since the risk of metachronous gastric cancer is long-lasting, long-term follow-up is required.

#### Treatments after eCuraC-1

Since the risk of harboring lymph node metastasis is low, one of the following alternatives could be selected according to the institutional policy after obtaining the patient’s consent: repeat ESD, surgical resection, close observation expecting a burn effect of the initial ESD, and endoscopic coagulation using a laser or argon-plasma coagulator [45].

When the lesion is differentiated type of ≤ 3 cm and one of UL1, pT1a (M) or pT1b1 (SM1), the size of the residual mucosal lesion should be reassessed by endoscopy. When the sum of the lengths of the resected and residual lesions exceeds 3 cm, gastrectomy with lymphadenectomy should be considered the standard of care. In addition, for patients with a positive horizontal margin within the portion of submucosal invasion and for those who underwent piecemeal resection in which the resection line involved the portion of submucosal invasion, gastrectomy with lymphadenectomy should be recommended, since the histological diagnosis under these circumstances is destined to be uncertain.

#### Treatments after eCuraC-2

Gastrectomy with lymphadenectomy should be considered the standard of care. When surgery cannot be recommended because of old age or severe comorbidities, the risk of residual disease in the form of lymph node metastasis (Tables 2 [46] and 3 [47]) and the possibility of subsequent local recurrence and/or distant metastasis should be assessed and explained sufficiently to the patient, along with the information that recurrent disease is usually incurable and has a dismal prognosis [48].

**Table 2:**
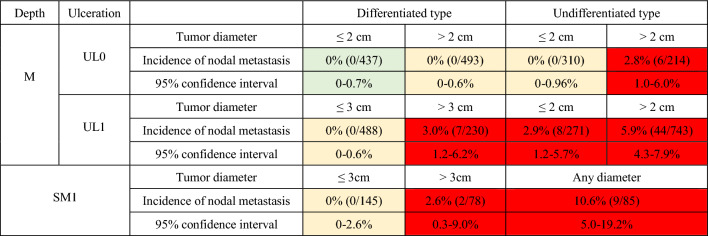
Incidence of nodal metastasis in various categories of early gastric cancer observed from surgically resected specimens operated at National Cancer Center Hospital and Cancer Institute Hospital [45]

The green zone indicates the absolute indication for endoscopic resection, the yellow zone indicates the expanded indication, and the red zone indicates the relative indication

**Table 3:** Incidence of nodal metastasis observed from the specimens of patients who underwent additional gastrectomy with lymphadenectomy after initial treatment with endoscopic resection

Total points	Number of patients (n = 1101)	Number of patients with lymph node metastasis (n = 94)	Incidence of nodal metastasis (%)(95% confidence interval)
0	62	1	1.6(0.0–8.7)
1	341	9	2.6(1.2–5.0)
2	185	9	4.9(2.3–9.0)
3	148	11	7.4(3.8–12.9)
4	132	11	8.3(4.2–14.4
5	141	28	19.9(13.6–27.4)
6	77	21	27.3(17.7–38.6)
7	15	4	26.7(7.8–55.1)

Total points refer to the total of the following scoring scheme, with one point added for each of the following findings: diameter ≥ 3 cm, positive vertical margin, venous invasion, and depth ≥ SM2. Three points are added for a histopathological finding of lymphatic invasion [46]

## Systemic chemotherapy for unresectable advanced/recurrent gastric cancer (AGC)

Advances in chemotherapy for AGC have significantly improved tumor shrinkage (response rates). In some cases such as those with microsatellite instability high (MSI-High) or HER2-positive disease, conversion surgery may be possible after incurable factors disappear by achieving remarkable efficacy of chemotherapy, and long-term survival may be expected. However, in most cases, it is difficult to obtain cure by chemotherapy alone. The median survival time achieved in domestic and international clinical trials for the disease at this stage remains about 17–18 months [49, 50]. The current goal of chemotherapy, therefore, is to delay the manifestation of, or ameliorate, disease-related symptoms and prolong survival.

Clinical benefits of chemotherapy have been proven in randomized, controlled trials comparing chemotherapy with best supportive care (BSC) in patients with performance status (PS) of 0–2, in terms of overall survival as the primary endpoint [51–53]. Although very rare, some patients with AGC actually survive more than 5 years. Thus, systemic chemotherapy is the treatment to be primarily considered for patients with AGC and those who have undergone non-curative (R2) resection.

### Principles of indications for systemic chemotherapy of AGC

Systemic chemotherapy is indicated for patients with AGC or those who have undergone R2 resection, provided their general condition and major organ functions are preserved. To be more specific, it is indicated for patients having PS 0–2 with either unresectable (locally advanced cancer and/or distant metastases) or recurrent gastric cancer.

#### Standard patient criteria for systemic chemotherapy

Before starting chemotherapy, the indication should be decided for each patient by checking the following items.

- Histologically proven gastric cancer
- PS 0–2. Chemotherapy is not generally recommended for patients with PS 3 or worse, and a decision beyond the scope of this recommendation should be made after discreetly considering the safety and clinical consequences for each patient (safety is of particular concern for AGC with massive ascites or extensive peritoneal metastases).
- Preserved major organ function
- No serious comorbidities
- Informed consent obtained from the patient

#### Routine evaluations before and during chemotherapy

1. The following should be checked or measured prior to initiation of chemotherapy: PS, body height, weight, symptoms, physical examination, laboratory data including hepatitis virus tests, and the size of tumor lesions assessed by computed tomography (CT) or other appropriate diagnostic modalities.
2. Response should be assessed using appropriate modalities such as CT, gastrointestinal endoscopy, and contrast-enhanced X-ray examination every 2 or 3 months, comparing the obtained images with those prior to initiation of chemotherapy or at the best response. Tumor response should be evaluated according to the Japanese Classification of Gastric Carcinoma or the Response Evaluation Criteria in Solid Tumors (RECIST) to decide whether to continue the ongoing chemotherapy.
3. The decision whether to continue the ongoing chemotherapy, to modify the drug dosage, or to change the treatment intervals should be made after carefully considering the adverse events and efficacy, and referring to the details of clinical trials showing the clinical significance of the treatment. Cumulative toxicities such as skin toxicities, dysgeusia, and peripheral neuropathy should be considered.
4. Appropriate management is needed for human hepatitis B virus carriers and infected patients to deliver chemotherapy according to the guidelines for reactivation of human hepatitis B virus (ref: https://www.jsh.or.jp/lib/files/medical/guidelines/jsh_guidlines/B_document-3_v2.pdf, in Japanese).

#### Anti-cancer agents

The following chemotherapeutic agents are proven to be beneficial for AGC: fluorouracil (5-FU), tegafur/5-chloro-2,4-dihydroxypyridine/potassium oxonate (S-1), levofolinate calcium, capecitabine, cisplatin, oxaliplatin, irinotecan, docetaxel, paclitaxel, nab-paclitaxel, trifluridine/tipiracil (FTD/TPI), trastuzumab, ramucirumab, nivolumab, pembrolizumab, trastuzumab deruxtecan, and zolbetuximab. These agents are used either as monotherapy or combination therapy based on the evidence obtained through clinical trials.

### Definition of the recommendation grade and evidence level for each chemotherapeutic regimen

The recommendation grade for each chemotherapeutic regimen in this section (Systemic chemotherapy for unresectable advanced/recurrent gastric cancer (AGC)) is classified into the following two levels, taking into consideration not only evidence from clinical studies, but also information from clinical practice in Japan.

#### Recommended regimens

In this guideline, recommended regimens are defined as those that fulfill one of the following requirements for patients whose general condition is sufficient to meet the inclusion criteria of clinical trials.

- Clinical utility, such as superiority over, or non-inferiority in terms of overall survival proven by a domestic or international phase III clinical trial.
- Reproducible clinical benefit for a specific patient population demonstrated by multiple domestic or international clinical trials.
- The regimen recognized as one of the standard regimens that has been adopted as a control arm in multiple domestic or international phase III clinical trials.

#### Conditionally recommended regimens

Conditionally recommended regimens are defined as those that fulfill one of the following requirements and can substitute for the “Recommended regimens” when deemed more appropriate after considering factors such as i) general condition of the patient including disease status, age, organ functions and comorbidities, ii) social factors such as hospitalization, cost of treatment, and distance to the hospital, and iii) personal preference that derives from the type of adverse events.

- Considerable clinical benefit under a specific condition in which the patient may not tolerate the “Recommended regimen”.
- Considerable clinical benefit based on wide usage in Japan in general practice or through interpretation of relevant clinical trials, even though the evidence is not robust enough to be included in the “Recommended regimens”.

**Fig. 8:**
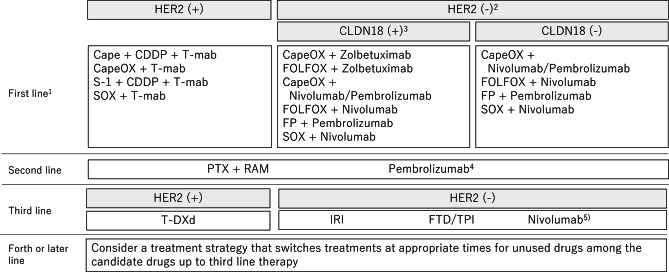
Recommended regimens for the first-, second-, third-, fourth-, or later-line treatments. Only the “Recommended regimens” as defined in the text are included. These regimens are recommended for patients who are in sufficiently good general condition to be eligible for the clinical trials from which the evidence in support of these regimens was generated. 1: Regimens are listed in alphabetical order. Oxaliplatin combination regimen is preferred. 2: Chemotherapy alone will be considered in cases with negative CLDN18 and low CPS. 3: The expression level of PD-L1 and the presence or absence of MSI-high/dMMR will be considered for administering nivolumab and pembrolizumab. 4: Pembrolizumab in second-line for MSI-High AGC is only recommended when nivolumab/pembrolizumab was not administered in first-line treatment. Weekly paclitaxel plus ramucirumab should be considered in third- or later-line treatment. 5: Nivolumab in third- or later-line treatment is not recommended when pembrolizumab or nivolumab was administered in previous treatment. S-1: tegafur/5-chloro-2,4-dihydroxypyridine/potassium oxonate, SOX: S-1 plus oxaliplatin, CDDP: cisplatin, T-mab; trastuzumab, CapeOX: capecitabine plus oxaliplatin, FOLFOX: fluorouracil/levofolinate calcium plus oxaliplatin, FP: fluorouracil plus cisplatin, PTX: paclitaxel, RAM: ramucirumab, T-DXd: trastuzumab deruxtecan, IRI: irinotecan, FTD/TPI: Trifluridine/tipiracil

**Fig. 9:**
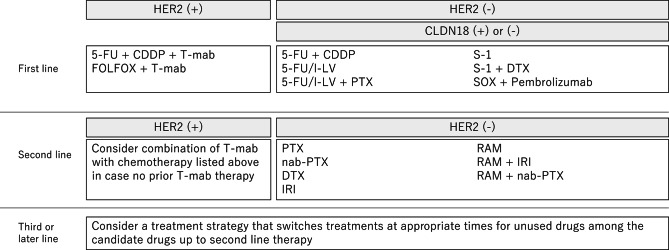
Conditionally recommended regimens shown in alphabetical order. CDDP: cisplatin, T-mab: trastuzumab, FOLFOX: fluorouracil/levofolinate calcium plus oxaliplatin, PTX: paclitaxel, DTX: docetaxel, nab-PTX: albumin-conjugated paclitaxel, IRI: irinotecan, RAM: ramucirumab

The “Recommended regimens” and “Conditionally recommended regimens” listed in Figs. 8 and 9 are selected based on voting by the seven medical oncologists who were members of the JGCA Guidelines Committee (the decision required agreement of at least 70% [5 of 7] of the medical oncologists). However, readers are not necessarily discouraged from using regimens that are not listed in these figures. The selection rule was stringent, and even the regimens that were supported by 50–69% [4 of 7] of the medical oncologists are not listed. Given the complexity of daily clinical practice, there could be situations where regimens that are not listed could, nevertheless, serve as useful options.

Due to the paucity of clinical trial results specific for elderly patients and for patients with impaired organ function or comorbidities, it is not possible to indicate with a sufficient evidence which is superior or safer, a “recommended regimen” delivered at a reduced dosage or modified treatment interval or a “conditionally recommended regimen”. Therefore, the optimal therapeutic regimen for these patients should be selected on a case-by-case basis, with the guidelines serving only as a reference. In addition, shared decision-making with patients is important when selecting a treatment method.

### First-line treatment for unresectable advanced/recurrent gastric cancer

In first-line chemotherapy of AGC, HER2 has been measured as a biomarker, and trastuzumab has been selected for HER2-positive cases. Currently, the anti-Claudin (CLDN)18.2 antibody zolbetuximab can be selected for CLDN18-positive cases. In addition, treatment including the anti-PD-1 antibody, nivolumab or pembrolizumab, has also been approved, and it has been reported that the presence or absence of MSI-High/mismatch repair deficient (dMMR) and the PD-L1 expression level (combined positive score: CPS) are related to the efficacy of these treatments. Therefore, the Japanese Gastric Cancer Association’s “Guidelines for Biomarker Testing for Unresectable Advanced and Recurrent Gastric Cancer” [54] recommend measuring these four biomarkers (HER2, CLDN18, PD-L1, MSI/MMR) before starting first-line chemotherapy, and it is desirable to select treatment based on the results (CQ13-1).

#### HER2-positive gastric cancer

Subgroup analysis of the ToGA study [55] showed that HER2 immunohistochemical staining (IHC) 3 + , or IHC2 + and in situ hybridization (ISH) positivity were more clearly associated with prolonged survival, and it is therefore recommended that patients with these findings be defined as HER2-positive for whom trastuzumab-containing chemotherapy is indicated. The frequency of HER2 positivity (IHC3 + or IHC2 + and ISH positive) in advanced and recurrent gastric cancer has been reported to be 17.8%. Drugs that can be used in combination with trastuzumab are fluoropyrimidine plus platinum. The capecitabine plus cisplatin in combination with trastuzumab regimen used in the ToGA trial, as well as S-1 plus cisplatin (SP), capecitabine plus oxaliplatin (CapeOX), and S-1 plus oxaliplatin (SOX) in combination with trastuzumab regimens, which showed reproducible efficacy in phase II trials, are “recommended” regimens [56–61] (CQ13-1).

#### HER2-negative gastric cancer

When the 6th edition of these guidelines was published in 2021, only HER2 was used as a biomarker for first-line chemotherapy for gastric cancer, and the recommended treatment for HER2-negative gastric cancer was combination therapy with fluoropyrimidine plus platinum.

Based on the results of the JCOG 9912 trial [62] and the SPIRITS trial [63] conducted in Japan, the S-1 plus cisplatin (SP) regimen was recommended. A capecitabine plus cisplatin (XP) regimen was also adopted as one of the standard treatments in the control groups of the ToGA trial [55] and the AVAGAST trial [64], and it was also recommended because its safety and efficacy were demonstrated in the subgroup analyses of Japanese cases in both trials. However, regimens using oxaliplatin, which is easier to administer than cisplatin because it does not require large-volume infusion, are also used. Capecitabine plus oxaliplatin (CapeOX) was shown to be effective in a subset analysis of a phase III trial in combination with epirubicin [65], and S-1 plus oxaliplatin (SOX) was shown to be almost as effective as SP in the G-SOX trial [49]. 5-FU/levofolinate calcium plus oxaliplatin (FOLFOX) therapy has been used as a control group treatment in recent comparative trials [66, 67] and is covered by insurance in Japan, so it may be an option. These combination therapies of fluoropyrimidine plus platinum were considered the “recommended” regimens for first-line chemotherapy until the previous edition of this guideline.

Currently, treatments that combine these chemotherapy regimens with nivolumab or pembrolizumab have been approved based on the results of multiple global phase III trials that included Japanese patients. The CheckMate649 trial [68] verified the superiority of CapeOX or FOLFOX plus nivolumab regimens, the ATTRACTION-04 trial [69] of SOX or CapeOX plus nivolumab regimens, and the KEYNOTE-859 trial [70] of FP or CapeOX plus pembrolizumab regimens over chemotherapy alone in terms of overall survival or progression-free survival.

Two global phase III trials have demonstrated the efficacy of combination therapy with chemotherapy and zolbetuximab in CLDN18-positive gastric cancer, and this treatment has been currently approved in Japan. The SPOTLIGHT trial [71] verified the superiority of mFOLFOX6 plus zolbetuximab therapy, and the GLOW trial [72] verified the superiority of CapeOX plus zolbetuximab therapy over the control treatment group (chemotherapy plus placebo).

Based on the above evidences, the combination of chemotherapy and immune checkpoint inhibitors, nivolumab or pembrolizumab, is recommended for first-line treatment of HER2-negative gastric cancer, and the combination of chemotherapy and zolbetuximab is recommended for CLDN18-positive cases. However, no clear research results comparing the efficacy of immune checkpoint inhibitors with zolbetuximab have been reported. It is necessary to consider that the therapeutic effects of nivolumab and pembrolizumab are strong in MSI-High/dMMR cases, whereas they tend to be weak in cases with low CPS values. The choice of chemotherapy alone in CLDN18-negative patients with low CPS levels, or combination therapy with immune checkpoint inhibitors or zolbetuximab in CLDN18-positive patients with high CPS levels, needs to be considered individually, taking into account factors such as the patient’s general condition, organ function, expected adverse events, and the patient’s wishes (CQ13-1).

### Second-line treatment for unresectable advanced/recurrent gastric cancer

Second-line treatment is recommended for patients with sufficient PS, because several randomized trials demonstrated a significant survival benefit of chemotherapy (irinotecan or docetaxel) over best supportive care (BSC) in the second-line setting [73–75].

The Japanese Phase III WJOG4007 trial compared irinotecan monotherapy with paclitaxel monotherapy, and the results showed good median survival times of approximately 9 months in both treatment arms [76]. Non-inferiority of weekly nab-paclitaxel (albumin-conjugated paclitaxel) monotherapy was proven over weekly paclitaxel monotherapy in the ABSOLUTE trial in Japan [77].

Since the paclitaxel plus ramucirumab combination was shown to be superior to weekly paclitaxel monotherapy in the phase III RAINBOW trial [78], this regimen is currently the “Recommended regimen”. In addition, the combination of nab-paclitaxel and ramucirumab is conditionally recommended because a certain degree of efficacy was demonstrated in a phase II study.

Furthermore, the REGARD trial [79] showed the survival benefit of ramucirumab monotherapy over BSC. Thus, monotherapy using any of the agents including paclitaxel, nab-paclitaxel, docetaxel, irinotecan, and ramucirumab is a “Conditionally recommended regimen” when the paclitaxel/ramucirumab combination is deemed unsuitable.

A use of biomarkers in the selection of second-line therapy is described in CQ13-2. The efficacy of continuing trastuzumab beyond progression for HER2-positive gastric cancer initially treated with a trastuzumab-containing regimen has been refuted by a randomized trial (WJOG7112G) [80], and it is strongly recommended not to continuously use trastuzumab beyond progression (CQ14). Although trastuzumab deruxtecan, which targets HER2, showed efficacy in third- or later-line treatments, its efficacy in second-line treatment is being investigated in ongoing clinical studies. At this point, the regimen in second-line treatment recommended for HER-2-positive gastric cancer is the same as that for HER2-negative gastric cancer.

Pembrolizumab is approved for patients with an unresectable advanced or recurrent MSI-high solid tumor that has progressed after chemotherapy. The frequency of MSI-high in advanced or recurrent gastric cancer is about 3–5%. The analysis of the KEYNOTE-158 trial, including gastric cancer patients with MSI-High, yielded relatively good response rates and progression-free survival of pembrolizumab monotherapy [81]. Pembrolizumab monotherapy in second-line treatment is recommended only for a case without prior use of immune checkpoint inhibitors.

### Third- or later-line treatment for unresectable advanced/recurrent gastric cancer

Third-line treatment should be considered after the end of second-line treatment, but a careful decision should be made regarding the indications for treatment. Monotherapy with nivolumab, irinotecan, or FTD/TPI is recommended for third- or later-line treatment for patients in good general condition. A clinical trial conducted in Korea comparing monotherapy with docetaxel or irinotecan versus BSC in second- and third-line treatments showed that chemotherapy extended survival, and its subgroup analysis of third-line treatment also suggested a survival benefit of chemotherapy over BSC [73]. Nivolumab and FTD/TPI both showed prolonged survival compared with placebo in patients with third- or later-line treatment in the ATTRACTION-2 trial [82] and the TAGS trial [83]. Based on the above, irinotecan, nivolumab, and FTD/TPI are recommended options for third-line treatment. However, since no study has directly compared nivolumab, irinotecan, and FTD/TPI, the priority and appropriate treatment sequence are not clear among these agents. Nivolumab is recommended only for a case without prior use of immune checkpoint inhibitors.

Treatment with trastuzumab deruxtecan, a drug-conjugated anti-HER2 antibody, showed a significantly higher response rate and longer overall survival than physician-chosen conventional chemotherapy (irinotecan or paclitaxel) in the randomized phase II trial (DESTINY-Gastric01 trial) [84] in Asia for HER2-positive gastric cancer patients who were treated with two or more prior regimens. In this trial, the combined alpha-error was set to be less than 0.05 at the two endpoints of response rate and overall survival, and it was below the significance level in the interim analysis. Although the number of cases was small, its survival benefit was verified statistically. Since trastuzumab deruxtecan is the only drug for which survival prolongation was confirmed in comparison with chemotherapy regimens in third-line treatment, trastuzumab deruxtecan is prioritized for third-line therapy of HER2-positive gastric cancer.

Even after completing third-line treatment, chemotherapy may be administered to patients in good general condition. Since there are few clear research results to select treatment options in this case, it may be appropriate to consider a treatment strategy in which a drug that has not been used previously among the candidate drugs up to third-line therapy is used at an appropriate time.

## Adjuvant chemotherapy

### Clinical significance of postoperative adjuvant chemotherapy

Postoperative adjuvant chemotherapy is delivered with the intention to reduce recurrence by controlling residual tumor cells after curative resection. As adjuvant chemotherapy, the efficacy of S-1 was proven in 2006 by the ACTS-GC trial [85, 86], a study that secured the place of postoperative S-1 monotherapy as a standard of care. The CLASSIC trial conducted in Korea, China, and Taiwan showed the prolongation of relapse-free survival by capecitabine plus oxaliplatin therapy (CapeOx) for TNM Stage II/III gastric cancer [87], and a study conducted in Japan confirmed its safety in Japanese patients [88]. A combination of S-1 and docetaxel was shown to have significant benefit for relapse-free survival over S-1 alone for Stage III gastric cancer in the JACCRO GC-07 trial [89].

In the phase III ARTIST2 trial of adjuvant chemotherapy for postoperative stage (pStage) II/III with D2 dissection in Korea, S-1 plus oxaliplatin (SOX) therapy significantly prolonged disease-free survival, the primary endpoint, compared with S-1 [90]. The additive effect of combining docetaxel or oxaliplatin in postoperative adjuvant chemotherapy was verified, but no additive effect was observed when combined with immune checkpoint inhibitors (ATTRACTION-5 trial) [91].

To conclude, adjuvant chemotherapy for pStage II/III gastric cancer is recommended because it was shown to improve survival after curative resection.

### Indications

One-year administration of S-1 for pStage II gastric cancer showed good clinical results (3-year relapse-free survival rate 93.1%, 3-year overall survival rate 96.1%) in the JCOG 1104 trial [92]. Together with the results of the ACTS-GC trial, 1-year postoperative adjuvant chemotherapy with S-1 is recommended for pStage II gastric cancer. However, S-1 monotherapy is a conditionally recommended regimen for pStage III gastric cancer, because combination therapies are recommended based on the results of the CLASSIC trial, JACCRO-GC07 trial, and ARTIST2 trial. Since S-1 plus docetaxel and CapeOX or SOX have not been directly compared among combination regimens, it is not possible to conclude which of these combination therapies is more effective at this time. It is important to select an appropriate regimen and maintain an appropriate dose and schedule, taking into consideration not only the pathological findings, but also the patient’s general condition and the occurrence of adverse events.

Postoperative chemotherapy for curatively resected Stage IV gastric cancer is weakly recommended because its effectiveness has been suggested retrospectively, but there is no evidence based on a prospective comparative clinical study (CQ16-2).

Several studies showed consistent results that chemotherapy for gastric cancer of CY1 after gastrectomy leading to macroscopically no residual tumor except for CY1 can achieve a 5-year survival rate of around 25%, although this is not strictly adjuvant chemotherapy (CQ5-4).

### Neoadjuvant chemotherapy

Neoadjuvant chemotherapy is premised on “curative resection” based on diagnostic imaging. It should be strictly distinguished from chemotherapy followed by surgery for borderline resectable cases and for initially unresectable cases converted to resectable due to its significant effect.

Though a wealth of experience in postoperative adjuvant chemotherapy has been accumulated in Japan, there are still not a few cases for whom it is difficult to perform intensive adjuvant chemotherapy due to decreased oral intake and complications. For these patients, the cure rate is expected to be improved by neoadjuvant chemotherapy, because it has the advantage of delivering intensive chemotherapy before surgery. Since postoperative adjuvant chemotherapy targets patients who have undergone curative resection, the indication can be accurately determined based on histological findings, but the indication for preoperative adjuvant chemotherapy is determined based on diagnostic imaging. A disadvantage of neoadjuvant chemotherapy is that some overdiagnosed cases who actually have early cancer not requiring peri-operative chemotherapy and underdiagnosed cases who actually have unresectable peritoneal metastases not detected by conventional imaging examination can be included as targets of neoadjuvant chemotherapy. There are also further disadvantages, such as the risk of becoming unresectable due to progression during chemotherapy and increased postoperative complications.

Recommendation for neoadjuvant chemotherapy should be made after carefully weighing these disadvantages against advantages. Thus, safety issues in terms of adverse events during chemotherapy and incidence of postoperative morbidity, accuracy of pretreatment staging, incidence of unresectable disease due to progression during neoadjuvant treatment, and QOL should be analyzed in future neoadjuvant trials in addition to proving superiority in survival outcomes over standard postoperative adjuvant therapies.

Neoadjuvant chemotherapy has been recognized as a standard of care in other countries, and the superiority of neoadjuvant chemotherapy has been reported in Western [93, 94], Chinese [95], and Korean [96] clinical trials. In Japan, favorable results have been reported by a phase II study for neoadjuvant chemotherapy with S-1 and cisplatin combination therapy for gastric cancer with “bulky N” status (CQ5-1), and this treatment is recognized as a tentative standard treatment for this particular population [97]. In addition, favorable pathological responses were reported for cases with advanced lymph node metastasis, including bulky N, when neoadjuvant chemotherapy was performed using the combination of S-1, oxaliplatin, and docetaxel [98]. However, no superiority of neoadjuvant chemotherapy with S-1 plus cisplatin was shown for large type 3 cancer and linitis plastica type gastric cancer [99].

Although perioperative adjuvant chemotherapy, including preoperative and postoperative chemotherapy, is considered the standard in other countries, there is no consensus on its introduction into daily medical practice in Japan because various problems have been identified (CQ16-1). It is important to clearly distinguish among resectable, borderline resectable, and unresectable, and to develop treatment strategies for each.

## Palliative care

Palliative care is an approach that improves the QOL of patients and their families facing the problems associated with life-threatening illness through the prevention and relief of suffering by means of early identification and careful assessment and treatment of pain and other problems, physical, psychosocial, and spiritual (WHO Definition of Palliative Care, 2002) [100].

The European Society for Medical Oncology defined ‘supportive care’ as care that aims to optimize the comfort, function, and social support of the patients and their family at all stages of the illness, and ‘palliative care’ as care when cure is not possible [101]. In recent years, the concept of patient-centered care, which integrated these two, has been proposed [102].

In Japan, supportive care is defined as ‘prevention, treatment, and care to reduce symptoms caused by cancer itself and side effects, complications, and sequelae associated with cancer treatment’ in the Basic Plan for Promotion of Cancer Control. Therefore, the definition of “palliative care and supportive care” is not the same inside and outside Japan. In addition, there is a large overlap between them, and it is appropriate to comprehensively consider it as “supportive/palliative medicine”.

This guideline 7th edition describes anamorelin for cachexia (CQ15-1) and radiation as palliative care for bleeding advanced gastric cancer (CQ15-2). Gastric cancer patients and their families also have various mental, social, and spiritual pains, in addition to physical pains, as do patients with other cancers. Palliative and supportive care for pain common to all these cancer treatments plays a basic role in cancer medical treatment. Many clinical studies are being conducted in the field of palliative and supportive care, mainly on pain management. All doctors who deal with cancer patients will need to acquire communication ability and learn not only about pain management but also about skills to support patients and relieve various types of pain and other symptoms (Fig. 9).

## Clinical pathway after surgery for gastric cancer

The enhanced recovery after surgery (ERAS) protocol is also widely used in gastric cancer, and its usefulness has been evaluated [103, 104]. The introduction of ERAS has been shown to enable early discharge. However, increased complications by accelerating the timing of oral intake have been reported [105], and it is necessary to consider the optimal timing at each facility. If there are no particular problems with the patient getting out of bed, it may be possible to initiate oral fluid intake on or after postoperative day 1, initiate solid food intake on or after day 2, and discharge on postoperative days 7–10 (Table 4).

**Table 4:** A common clinical pathway for distal, total, and proximal gastrectomies

Clinical item	Day of the clinical pathway
Removal of nasogastric tube	Before or on postoperative day 1
Initiation of oral fluid intake	On or after postoperative day 1
Initiation of solid food intake	Postoperative days 2–4
Prophylactic administration of antibiotics	Only on the day of operation
Removal of epidural tube	Before or on postoperative day 3
Removal of urinary catheter	Before or on postoperative day 3
Intravenous fluid administration	Until postoperative days 5–7
Removal of intra-abdominal drains	Before or on postoperative day 5
Discharge from the hospital	Postoperative days 8–14

## Follow-up surveillance after surgery for gastric cancer

**Fig. 10:**
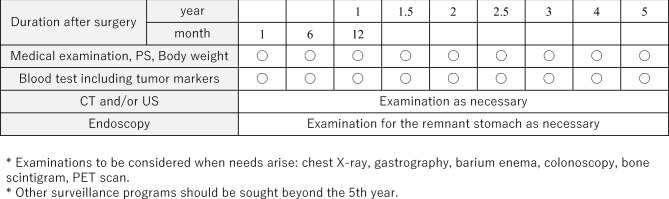
Postoperative follow-up for Stage I gastric cancer patients

**Fig. 11:**
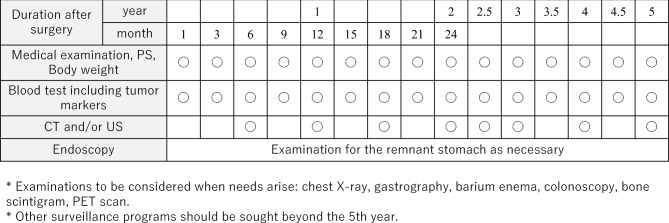
Postoperative follow-up for Stage II–III gastric cancer patients

Life guidance after gastrectomy and treatment for post-gastrectomy syndrome are provided, and follow-up is systematically performed according to the risk of recurrence for early detection of recurrence and secondary cancer. There is little evidence that it can be expected to prolong survival [106, 107]. In addition, since there is no prospective research on regular postoperative follow-up methods, there is little evidence of appropriate follow-up tests and follow-up intervals. However, CT, tumor markers (CEA + CA19-9), and endoscopy are thought to be useful to detect recurrence, remnant tumor, and double cancer judging from some retrospective studies. Tumor markers are elevated at the time of recurrence and may precede diagnostic imaging findings by about 2 or 3 months [108]. Based on the results of recurrence/relapse time, follow-up as shown in Fig. 10 for early-stage cancer and Fig. 11 for advanced cancer is presented for reference.

In principle, follow-up for 5 years after surgery is required. However, since recurrence or metachronous multiple cancer may be discovered after 5 years [109], users should individually decide the effective use of not only the users’ own facility, but also referring doctors, collaborating doctors, basic medical examinations, workplace examinations, and comprehensive medical checkups. The contribution of the planned follow-up after surgery to patients’ survival should be scientifically validated in the future.

## Clinical questions for surgery (Table 5)

**Table 5:** Definition of the evidence level

Strength of the body of evidence	
A (strong)	Strong reliability in the expected value of the effect
B (moderate)	Moderate reliability in the expected value of the effect
C (modest)	Limited reliability in the expected value of the effect
D (weak)	Almost not reliable for the expected value of the effect

### Clinical questions for the indication of minimally invasive surgery

#### CQ1-1 Is laparoscopic gastrectomy recommended for resectable gastric cancer? (Please refer to CQ1-2 for robotic gastrectomy and CQ1-3 for laparoscopic total gastrectomy for advanced gastric cancer)

**Recommendation**

Laparoscopic distal gastrectomy is strongly recommended as one of the standard treatments (Consensus rate 100%, 10/10, Strength of evidence A). Laparoscopic total gastrectomy or proximal gastrectomy for cStage I gastric cancer is strongly recommended (Consensus rate 78%, 7/8, Strength of evidence C). The procedures must be conducted by a qualified surgeon in the endoscopic surgical skill qualification system of the Japanese Society of Endoscopic Surgery or a surgeon with equivalent skills or under the guidance of an instructor with equivalent skills.

#### CQ1-2 Is robotic gastrectomy recommended for resectable gastric cancer?

**Recommendation**

Robotic gastrectomy is weakly recommended for resectable gastric cancer.

The procedures should be performed under the guidelines of the Japanese Society of Endoscopic Surgery and insurance approval requirements (Consensus rate 100%, 10/10, Strength of evidence C).

#### CQ1-3 Is laparoscopic total gastrectomy recommended for advanced gastric cancer?

**Recommendation**

Laparoscopic total gastrectomy is weakly recommended as a standard treatment. The procedures must be conducted by a qualified surgeon in the endoscopic surgical skill qualification system of the Japanese Society of Endoscopic Surgery or a surgeon with equivalent skills or under the guidance of an instructor with equivalent skills (Consensus rate 90%, 9/10, Strength of evidence C).

#### CQ1-4 Is minimally invasive surgery (laparoscopic or robotic) recommended for patients who underwent preoperative chemotherapy?

**Recommendation**

Minimally invasive surgery is weakly recommended for patients who received preoperative chemotherapy. The procedures must be conducted by a qualified surgeon in the endoscopic surgical skill qualification system of the Japanese Society of Endoscopic Surgery or a surgeon with equivalent skills or under the guidance of an instructor with equivalent skills (Consensus rate 100%, 10/10, Strength of evidence C).

### Clinical questions for function-preserving surgery

#### CQ2-1 Is pylorus-preserving gastrectomy (PPG) recommended for early gastric cancer in the middle portion of the stomach?

**Recommendation**

PPG is weakly recommended for early gastric cancer in the middle portion of the stomach (Consensus rate 100%, 10/10, Strength of evidence C).

#### CQ2-2 Is proximal gastrectomy recommended for early proximal gastric cancer as function-preserving surgery?

**Recommendation**

Proximal gastrectomy is weakly recommended for early proximal gastric cancer as function-preserving surgery (Consensus rate 90%, 9/10, Strength of evidence C).

#### CQ2-3 Is subtotal distal gastrectomy with minimal preservation of the fundus recommended for cancer of the upper third of the stomach?

**Recommendation**

If an adequate resection margin can be ensured, subtotal distal gastrectomy with minimal preservation of the fundus is recommended for cancer of the upper third of the stomach (Consensus rate 100%, 10/10, Strength of evidence C).

### Clinical questions for combined resection and extended surgery

#### CQ3-1 Is omentectomy recommended for advanced gastric cancer?

**Recommendation**

Omentectomy is weakly recommended for cT3-T4 gastric cancer (Consensus rate 80%, 8/10, Strength of evidence C).

#### CQ3-2 Is splenectomy or splenic hilar lymph node dissection recommended for advanced proximal gastric cancer?

**Recommendation**

It is strongly recommended not to perform splenectomy or splenic hilar lymph node dissection for tumors which have not invaded the greater curvature (Consensus rate 100%, 9/9, Strength of evidence A). Splenectomy or splenic hilar lymph node dissection is weakly recommended for tumors which have not invaded the greater curvature (Consensus rate 90%, 9/10, Strength of evidence C).

#### CQ3-3 Is pancreaticoduodenectomy recommended in advanced gastric cancer with invasion of the duodenum or head of the pancreas?

**Recommendation**

Pancreaticoduodenectomy is weakly recommended for advanced gastric cancer with invasion of the duodenum or head of the pancreas with certain conditions (Consensus rate 100%, 10/10, Strength of evidence C).

### Clinical questions for appropriate staging

#### CQ4-1 Is PET-CT recommended for the staging of gastric cancer?

**Recommendation**

It is weakly recommended not to perform PET-CT (Consensus rate 70%, 7/10, Strength of evidence C).

#### CQ4-2 Is staging laparoscopy recommended for determining the treatment strategy in advanced gastric cancer?

**Recommendation**

Staging laparoscopy is weakly recommended to determine the treatment strategy for patients with advanced gastric cancer who are likely to have peritoneal metastasis (Consensus rate 90%, 9/10, Strength of evidence B).

### Clinical questions for Stage IV gastric cancer

#### CQ5-1 Is surgical treatment for oligometastases recommended?

**Recommendation**

Surgical resection after neoadjuvant chemotherapy is weakly recommended for a small number of paraaortic lymph node metastases confined to No.16a2/b1, if there are no other non-curable factors (Consensus rate 94.7%, 18/19, Strength of evidence C).

Surgical resection is weakly recommended for solitary liver metastasis, if there are no other non-curable factors (Consensus rate 100%, 19/19, Strength of evidence C).

#### CQ5-2 Is conversion surgery (including postoperative adjuvant chemotherapy) recommended?

**Recommendation**

At present, it is difficult to make a clear recommendation regarding conversion surgery for Stage IV patients due to a lack of evidence (Consensus rate 78.9% (15/19), Strength of evidence C).

In addition, there are no clear recommendations regarding adjuvant chemotherapy for Stage IV gastric cancer patients in whom R0 resection has been achieved through conversion surgery (Consensus rate 78.9% (15/19), Strength of evidence C).

#### CQ5-3 Is palliative resection, bypass surgery, or stent placement recommended for bleeding or stenosis from incurable gastric cancer?

**Recommendation**

Palliative resection, bypass surgery, or stent placement is weakly recommended for bleeding or stenosis from incurable gastric cancer (Consensus rate 100%, 19/19), Strength of evidence D).

#### CQ5-4 Is gastrectomy recommended for CY1? (Including postoperative chemotherapy)

**Recommendation**

If CY1 is identified during gastrectomy, upfront gastrectomy followed by postoperative chemotherapy is weekly recommended (Consensus rate: 94.7% 18/19, Strength of evidence C).

If CY1 is identified before the initial treatment by staging laparoscopy, gastrectomy is weakly recommended when CY0 is achieved after chemotherapy (Consensus rate 100%, 19/19, Strength of evidence C).

### Clinical questions for esophagogastric junctional cancer

#### CQ6-1 Is mediastinal lymph node dissection or para-aortic lymph node dissection recommended for the surgery for esophagogastric junctional cancer?

**Recommendation**

In surgery for esophagogastric junctional cancer deeper than cT2,

1. Lower mediastinal lymph node dissection is weakly recommended if the esophageal invasion length is greater than 2 cm (Consensus rate 90%, 9/10, Strength of evidence C).
2. Upper, middle, and lower mediastinal lymph node dissection is weakly recommended if the esophageal invasion length is greater than 4 cm (Consensus rate 80%, 8/10, Strength of evidence C).
3. It is weakly recommended not to perform para-aortic lymph node (No.16a2) dissection, taking into consideration the entire surgical stress (Consensus rate 100%, 10/10, Strength of evidence C).

#### CQ6-2 Is proximal gastrectomy recommended for esophagogastric junctional cancer?

**Recommendation**

Proximal gastrectomy is weakly recommended for esophagogastric junctional cancer (Consensus rate 100%, 10/10, Strength of evidence C).

#### CQ6-3 Is laparoscopic or robotic surgery recommended for esophagogastric junctional cancer?

**Recommendation**

Laparoscopic or robotic surgery is weakly recommended for esophagogastric junctional cancer (Consensus rate 70%, 7/10, Strength of evidence D).

### Clinical questions for carcinoma of the gastric remnant

#### CQ7-1 Is lymph node dissection with splenectomy recommended for carcinoma of the gastric remnant?

**Recommendation**

Splenic hilar dissection with splenectomy is weakly recommended for advanced carcinoma of the gastric remnant with greater curvature invasion (Consensus rate 100%, 10/10, Strength of evidence C).

Not performing splenic hilar lymph node dissection with splenectomy for tumors without greater curvature invasion is weakly recommended (Consensus rate 80%, 8/10, Strength of evidence C).

#### CQ7-2 Is laparoscopic or robotic surgery recommended for carcinoma of the gastric remnant?

**Recommendation**

At present, it is difficult to make a clear recommendation to perform laparoscopic or robotic surgery for carcinoma of the gastric remnant (Consensus rate 70%, 7/10, Strength of evidence D).

#### CQ7-3 Is mesenteric lymph node dissection recommended for carcinoma of the gastric remnant located at the gastrojejunostomy

**Recommendation**

Mesenteric lymph node dissection is weakly recommended for carcinoma of the gastric remnant located at the gastrojejunostomy (Consensus rate 100%, 10/10, Strength of evidence C).

### Clinical question for reoperation after gastrectomy

#### CQ8 Is reoperation recommended if the resection margin is diagnosed as positive in the permanent section?

**Recommendation**

It is difficult to make a clear recommendation for reoperation if the resection margin is diagnosed as positive in the permanent section (Consensus rate 100%, 10/10, Strength of evidence D).

### Clinical questions for long-term impairment after gastrectomy

#### CQ 9–1 Is vaccination with pneumococcal vaccine recommended after splenectomy?

**Recommendation**

Pneumococcal vaccination after splenectomy for gastric cancer surgery is weakly recommended (Consensus rate 90%, 9/10, Strength of evidence D).

#### CQ9-2 Is administration of vitamin B12 recommended after total gastrectomy?

**Recommendation**

Administration of vitamin B12 is weakly recommended after total gastrectomy (Consensus rate 90%, 9/10, Strength of evidence C).

#### CQ9-3 Is *Helicobacter pylori* eradication recommended after gastrectomy?

**Recommendation**

Considering the prolongation of survival, lower recurrence rate, lower incidence of metachronous gastric cancer, increase in serious adverse events, increase in adverse events (including minor ones), and increase in costs, there is no clear recommendation for *Helicobacter pylori* eradication therapy after gastrectomy (Consensus rate 100%, 11/11, Strength of evidence C).

### Clinical questions for elderly patients

Clinical questions regarding surgery, chemotherapy, and endoscopic resection for elderly gastric cancer patients.

#### CQ10-1 Is considering age recommended when deciding a surgical procedure?

**Recommendation**

Limited surgery with reduced lymph node dissection or minimally invasive surgery is weakly recommended for elderly patients (Consensus rate 100%, 19/19, Strength of evidence D).

#### CQ10-2 Is considering age recommended when deciding on the indication for systemic chemotherapy?

**Recommendation**

Considering age when deciding on the indication of systemic chemotherapy is weakly recommended for an elderly patient with unresectable advanced or recurrent gastric cancer, who is fit, has decision-making capacity, and is willing to undergo treatment after carefully assessing the patient’s overall condition and motivation (Consensus rate 100%, 19/19, Strength of evidence B).

In other cases, such as vulnerable or unfit patients, no clear recommendation can be made due to the range of the condition.

#### CQ10-3 Is considering of age recommended when deciding on the indication of endoscopic resection?

**Recommendation**

There are no reports directly comparing ESD with and without considering age in elderly patients with early gastric cancer, nor reports comparing observation with ESD. A recommendation regarding considering age when deciding on the indication for endoscopic resection cannot be made (Consensus rate 73.7%, 14/19, Strength of evidence D).

#### CQ10-4 Is perioperative nutritional/exercise therapy recommended for elderly patients or patients with sarcopenia?

**Recommendation**

No clear recommendations can be made regarding perioperative nutritional/exercise therapy for elderly patients or patients with sarcopenia (Consensus rate 94.7%, 18/19, Strength of evidence D).

### Clinical questions for chemotherapy

Clinical questions regarding chemotherapy for unresectable advanced/recurrent gastric cancer (AGC).

#### CQ11-1 Is chemotherapy recommended for patients with impaired oral intake or massive ascites due to extensive peritoneal disease?

**Recommendation**

Chemotherapy is weakly recommended for patients with impaired oral intake or massive ascites after careful assessment of their general condition (Consensus rate 100%, 7/7, Strength of evidence C).

#### CQ11-2 Is personalized medicine based on genomic profiling tests recommended for AGC?

**Recommendation**

It is weakly recommended to perform appropriate genomic profiling tests in later-line treatment and to treat based on the genetic alterations found for unresectable advanced or recurrent gastric cancer patients (Consensus rate 100%, 7/7, Strength of evidence C).

#### CQ12 Are immune checkpoint inhibitors recommended for first-line treatment of HER2-negative AGC?

**Recommendation**

Chemotherapy in combination with an immune checkpoint inhibitor (nivolumab or pembrolizumab) is strongly recommended as first-line treatment for HER2-negative unresectable advanced/recurrent gastric/gastroesophageal junction cancer, taking into account biomarkers (PD-L1 (CPS), MSI, MMR, CLDN18) and the patient’s general condition (Consensus rate 100%, 7/7, Strength of evidence A).

#### CQ13-1 Is selection of first-line treatment based on biomarkers recommended?

**Recommendation**

Selection of first-line treatment based on biomarkers is strongly recommended for patients with unresectable advanced or recurrent gastric cancer (Consensus rate 100%, 7/7, Strength of evidence A).

#### CQ13-2 Is it recommended to select second-line or later treatments based on biomarkers obtained on pretreatment repeat biopsy?

**Recommendation**

No clear recommendations can be made regarding second-line or later treatment selection based on biomarker (HER2) testing by repeat biopsy for patients with unresectable advanced gastric or gastroesophageal junction adenocarcinoma (Consensus rate 85.7%, 6/7, Strength of evidence D).

#### CQ14 After progression of chemotherapy for unresectable advanced or recurrent gastric cancer, is it recommended to continue or re-administer drugs used in previous treatment?

**Recommendation**

It is strongly recommended not to continuously use or re-administer fluoropyrimidine, trastuzumab, or ramucirumab in chemotherapy for unresectable advanced or recurrent gastric cancers (Consensus rate 100%, 7/7, Strength of evidence A).

### Clinical questions for palliative care

#### CQ15-1 Is anamorelin recommended for cachexic patients?

**Recommendation**

In patients with advanced gastric cancer and a non-refractory cachexia state, appropriate dosing of anamorelin is weakly recommended to promote appetite and weight gain (Consensus rate 100%, 7/7, Strength of evidence D).

#### CQ15-2 Is radiation recommended as palliative treatment for bleeding advanced gastric cancer?

**Recommendation**

For patients with incurable advanced gastric cancer with bleeding, radiation for the purpose of hemostasis is weakly recommended, taking into consideration the patient’s overall condition and prognosis (Consensus rate 100%, 7/7, Strength of evidence C).

### Clinical questions for perioperative chemotherapy

#### CQ16-1 Is neoadjuvant chemotherapy for curatively resectable advanced gastric and esophagogastric junctional cancer recommended?

**Recommendation**

No clear recommendations can be made regarding neoadjuvant chemotherapy for curatively resectable advanced gastric and esophagogastric junctional cancer (Consensus rate 89.5%, 17/19, Strength of evidence C).

#### CQ16-2 Is adjuvant chemotherapy recommended for stage IV gastric cancer with R0 resection?

**Recommendation**

Adjuvant chemotherapy is weakly recommended for stage IV gastric cancer with R0 resection (Consensus rate 94.7%, 18/19, Strength of evidence C).

### Clinical questions for endoscopic resection

#### CQ17 Is continuation of antithrombotic drugs recommended compared with temporary discontinuation of the drugs when performing endoscopic resection of patients taking antithrombotic drugs?

**Recommendation**

No recommendation can be made regarding the usefulness of continuation of antithrombotic drugs compared with temporary discontinuation of the drugs when performing endoscopic resection of patients taking antithrombotic drugs.
